# HIV-positive patients’ perceptions of care received at a selected antiretroviral therapy clinic in Vhembe district, South Africa

**DOI:** 10.4102/phcfm.v8i2.926

**Published:** 2016-04-26

**Authors:** Tshifhiwa V. Ndou, Sonto M. Maputle, Patrone R. Risenga

**Affiliations:** 1 Department of Advanced Nursing, University of Venda, South Africa; 2 Department of Health Studies, University of South Africa, South Africa

## Abstract

**Background:**

Patients’ experiences are a reflection of what has happened during the care process and, therefore, provide information about the performance of health care professional workers. They refer to the process of care provision at the antiretroviral therapy (ART) sites.

**Aim and setting:**

This article explored the perceptions of HIV-positive patients of care received at the Gateway Clinic of the regional hospital that provides antiretroviral treatment in the Vhembe district.

**Methods:**

A qualitative, explorative and descriptive design was used. A non-probability, convenient sampling method was used to select 20 HIV-positive patients who were above 18 years of age. In-depth individual interviews were used to collect data. Data were analysed through Tech’s open coding method.

**Results:**

One theme and two sub-themes emerged, namely positive experiences related to the environment and attitudes of health professionals, and negative experiences concerning the practices by health care providers.

**Conclusion:**

Patients’ perceptions of quality of, and satisfaction with, health care may affect health outcomes. Recommendations are made to consider, practice and strengthen the protocols, the standard operating procedures and the principles of infection control in the health facilities.

## Introduction

Patient experiences can be defined as reflections of what has actually happened during the care process and, therefore, provide information about the performance of health care professional workers. Jenkinson et al.^1^ refers to the process of care provision at the antiretroviral therapy (ART) sites.^2^ Since the 1990s, the introduction of Highly Active Antiretroviral Therapy (HAART) has modified the clinical course of HIV infection, reducing the rate of disease progression, the incidence of opportunistic infection and mortality.^3^ Despite the remarkable improvements in the HIV treatment and prevention, economic and social barriers that result in continued morbidity, mortality, and HIV infection persist. ART is to be delivered through health centres as part of a package of care that includes co-trimoxazole prophylaxis, counselling, the management of opportunistic infections and comorbidities, provision of antiretroviral drugs and nutritional support. Yoder et al.^4^ indicated that it is important to understand the experiences and perceptions of care by HIV-positive patients who are taking medications in order to provide the needs-specific care to patients. Although satisfaction has been observed with regard to HIV services and management, Kieft et al.^5^ indicated that less is known about perceptions of clinical care, particularly for HIV-positive patients.

Liau et al.^6^ identified five steps in the HIV Care Continuum required for reaching undetectable viral loads for patients infected with HIV, namely initial HIV diagnosis, linkage to care, retention in care, receipt of ART, and achievement of a low viral load at an undetectable level. Zuidgeest et al.^7^ said that assessing patients’ perceptions of the quality of care not only provides information about the actual experiences but also reveals which quality aspects patients regard as most important. HIV-positive patients often encounter health systems which are ill-equipped to meet their needs effectively.^8^ This may result in the HIV-positive patient missing doses or dropping out. The attention to physical and environmental needs of the client is very important. Hence, Ehlers^9^ was of the opinion that there has been little examination of the needs, expectations and interactions of ART patients and the nurses who care for them. The understanding of what patients and nurses perceive to be good ART-related clinical care and exploring differences in these perceptions is a vital component of improving HIV care in poor countries. Most studies on patient-health worker interaction in the field of HIV focus on adherence issues.^9^ Improving the clinical experiences for patients and health staff in resource-poor environments is vital to support the on-going response to HIV, because good quality patient-health worker relationships promote adherence. This study explored the perceptions of HIV-positive patients of care received at selected ARV clinics in Vhembe district, South Africa.

### Purpose

The purpose of the study was to determine the perceptions of HIV-positive patients of care provided by health professionals at the ART clinic of the regional hospital in Vhembe district.

### Objective

The objective was to explore and describe the perceptions of HIV-positive patients of care provided by health professionals at the ART clinic of the regional hospital in Vhembe district.

## Research methods and design

### Study design

The study was conducted at the ART clinic of the regional hospital in Vhembe district of the Limpopo Province, South Africa. This hospital was selected because it is a referral hospital for all hospitals within the district. A qualitative explorative and descriptive design was used to explore the perceptions of HIV-positive patients with regard to care received at the ARV sites in Vhembe district.

### Population and sampling strategy

The population consisted of both male and female HIV-positive patients who are on antiretroviral treatment at ART clinic of Tshildzini hospital in the Vhembe district of Limpopo Province, South Africa. Non-probability, convenient and purposive sampling was used to select participants. The inclusion criteria were that participants should be HIV-positive, on ARV treatment, above the age of 18 years, willing to participate in sharing their knowledge and prepared to sign an informed consent form. Twenty participants, 5 males and 15 women, participated in the study.

### Data collection

Ethical clearance was obtained from the Ethics Committee of the University of Venda (SHS/11/PDC/04/E1011). The Provincial Department of Health gave permission to access the health facility and conduct the study. Data were collected by means of in-depth individual, unstructured interviews.^10,11^ An appointment for an interview was secured with the client before he or she left the clinic. The interview focused on perceptions of HIV-positive clients of care received at the ARV facility in Vhembe district. The question which guided the interview was: ‘Could you kindly share with me: how did you experience the care provided by health care professionals at this clinic?’ The question was followed by probing as a communication skill, which elicited more information from the participants as postulated by De Vos et al.^10,12,13^ The interviews were conducted in the local language (Tshivenda) by the researchers in a private cubicle at the clinic for about 45 min to one hour. Permission to use a voice recorder was obtained, and recordings were transcribed verbatim and translated into English.^10^ Data were collected from participants until data saturation was reached.^14^

### Data analysis

The narrative data from the in-depth interviews were analysed qualitatively using Tesch’s open coding method cited in Creswell.^15^ The method included the following steps: the researcher read carefully through all the transcripts to gain an understanding of the data. After the completion of all transcripts, a list of similar topics was compiled. Data were grouped according to a theme and sub-themes and field notes were also coded and categorised. A literature control was done to contextualise the results of the study.^15^ To ensure trustworthiness, criteria as outlined in Lincoln and Guba^16^ were used. Credibility was ensured by prolonged engagement to increase rapport and clarify descriptions with participants through familiarity, and participants were able to talk freely about more hidden and sensitive information.^17^ Data triangulation was ensured by using different data collection methods, namely in-depth individual interviews, voice recorder and field note-taking. Member checking was done to confirm and validate the findings through interviews and discussion with colleagues in order to discover the truth.

## Results

The total number of participants was 20: 15 females and 5 males of the ages between 29 and 58 years. Fifteen participants were married, five were single, and all participants had children. Participants were HIV-positive and were already on antiretroviral treatment for the period of six months and longer. The participants were able to express themselves clearly in their language as researchers were able to understand and talk Tshivenda as well as English. The participants’ educational level ranged from Standard 5 and above Grade 12 (Grade 7 – Tertiary education).

The theme and sub-themes which reflect the perceptions of HIV-positive patients on care received at ARV clinic emerged during data analysis, as summarised in Table 1.

**TABLE 1:** Theme and sub-themes as perceptions of care received at a selected antiretroviral therapy clinic.

Theme	Sub-themes
Health care providers’ practices at ART clinic.	1.1 Positive experiences related to the environment and attitudes of health professionals. 1.2 Negative experiences with regard to the practices by healthcare providers.

*Source*: Author’s own work antiretroviral therapy

### Theme: Health care providers’ practices at antiretroviral therapy clinic

Participants had both positive and negative experiences when they received care or treatment from the ART clinic; for example, they indicated that nurses explained procedures to them and they knew who to contact if they experienced problems. Shaller^18^ was of the opinion that patient care requires well-trained nurses who are capable of creating a safe and patient-centred environment. The physical environment of an AIDS-designated unit may be less significant than the competent care demonstrated by the nurses who are employed on those units.^5^ Ding et al.^19^ suggested that care can be poor even at sites with good support services if there is not a suitable provider relationship. The effective use of medical and support services probably requires a provider who knows each patient and can facilitate the provision and integration of care.

Under this theme, the following sub-themes emerged, namely positive experiences related to the environment and attitudes of health professionals, and negative experiences with regard to the practices by health care providers. The sub-themes are discussed below.

### Sub-theme 1.1: Positive perceptions related to the environment and attitudes of health professionals

Patients’ perceptions about the quality level of health services provided in hospitals seem to have been largely ignored by both researchers and practitioners. Patients’ voices have to guide the design of health care service delivery processes in order to foster confidence and promote the usage of available health care facilities. The attention to physical and environmental needs is very important.

Positive remarks were made by the participants in regarding the cleanliness of the environment, which is maintained by health care providers at the ART clinic. Participant 5 (female, 58 years, Grade 7 and unemployed) confirmed this by saying: ‘At this ART clinic, we feel very safe and cared for. The rooms are clean. They are always clean, chairs dusted all the time.’ The participants in this study showed how clean the counselling rooms and pharmacy were. Participant 5 (female, 58 years, Grade 7 and unemployed) said: ‘I always find that the wellness care clinic is clean and the risk of getting infection here is low, we are safe.’

It is of crucial importance that the health care facilities are kept clean for the health of the HIV-positive patients on ART. According to the Standard Operating Procedure of the Ministry of Health,^20^ HIV counselling rooms should ensure privacy; people outside the room should not hear voices inside the room and the rooms should be closed. The rooms should be free from dirt, smoke or anything such as water spilling from washbasins.^21^

Participants were of the opinion that health providers were friendly and encouraging as they (the patients) were discouraged because of their AIDS diagnosis. Participants reported their acceptance by the staff irrespective of their HIV-positive status. They felt that nurses’ encouraged them to take AIDS as any other chronic illness that could be managed and controlled by ART. This concurs with the findings by Kieft et al.^5^ who indicated that the HIV-positive women and minority participants were generally satisfied with their hospital care. The physicians, nurses, and the hospital environment received satisfactory ratings.

Some of the participants reported staff attitudes of acceptance and friendliness towards the HIV-positive patients on ART. The following quotes illustrate this:

‘They accepted me as who I am. They told me to view ART as any other treatment for other chronic illness like hypertension and diabetes which are also taken daily for a lifetime period.’ (Participant 8, female, 30 years, post-secondary school education and employed)

Participant 1 (male, 48 years, Grade 9 and unemployed) experienced a similar attitude by the nursing staff:

‘The nurses were friendly and informative about how ARVs work when taken and this made me to adhere to taking my treatments. They just tell me that ART is like any other medications for chronic illness and were friendly towards me.’

Participant 10 (male, 50 years, Grade 10 and unemployed) was grateful for the staff attitude:

‘The staff was friendly towards me as a patient. They accepted me and encouraged me to continue taking treatment, they even show me others who are sick like me and are now well I felt encouraged and grateful.’

Attitude has been one of the central areas of study on HIV and AIDS due to the impact it has on the health service. The study findings of Aghamolaei et al.^22^ revealed that the health care providers had positive attitudes towards patients with HIV. This was supported by Stein,^23^ who revealed that the HIV and AIDS stigma has already diminished substantially over time both in South Africa and worldwide due to ART. Two surveys conducted in South Africa identified a decreasing HIV and AIDS stigma in the general population. Furthermore, Stein^23^ stated that South Africans are adopting accepting attitudes towards people living with HIV and AIDS. The researcher in this study found that the participants experienced health care providers and family as encouraging.

Participants also related stories that showed they were experiencing problems with regard to safety measures in the ART clinic. The following quote illustrates what Participant 2 (female, 32 years, post-secondary education and employed) said:

‘On arrival before I am checked, the nurses will check my name when issuing the medicines and ask if what they giving me are the correct medication which I usually receive.’

This was supported by Participant 4 (female, 57 years, Grade 8 and unemployed), who said:

‘I was never given the expired medication. I have never heard that anyone fell due to slippery floors. Sometimes when you want to pass the cleaners will ask you to use another way or put a warning showing it is wet. The staff here are very cautious.’

Other participants mentioned that the health care staff observed the safety requirements for storing sharp objects:

‘I am happy, were are safe here … they put all needles and waste away free of reach from the patients.’ (Participant 9, female, 29 years, Grade 11 and unemployed)

Aghamolaei et al.^22^ argued that, in order for medical facilities to effectively secure patients’ safety, they should identify and eliminate known hazards by using adequate cleaning and disinfectant procedures and standardised measures for needle and waste disposal. In this study, participants felt safe and they showed how needle and waste disposal measures were used.^24^ The perception of care and positive relationships with health providers is important to participants, and these seemed to be based on approachability and empathy.

### Sub-theme 1.2: Negative experiences with regard to the practices by health care providers

Heyer et al.^25^ were of the opinion that people living with HIV and AIDS (PLWHA) face challenges and usually experience symptoms like anxiety, shame with a shattered sense of hope and life purpose and control of self-worth. One participant in their study related how he experienced negative attitudes from the nursing staff whilst he attended the ARV clinic, because he was diagnosed of AIDS stage 4:

‘When I was too ill, and was brought to the clinic, I felt very much isolated as the nurse put on more gloves when coming to examine me whereas on handling other patients he only put on one pair. I did not like to associate with other patients because of different treatment.’

Manala^26^ described how a patient in a hospital was isolated in a separate ward; staff were afraid to touch him because he had HIV and AIDS. A study conducted by Aghamolaei et al.^22^ revealed that most health care providers showed positive attitudes towards patients with HIV and who were taking ART. However, those who showed negative attitudes reported fear of infection as the major problem. Health care providers need to be notified if a patient has HIV and is taking ART so that they can apply better protective measures against infection. According to Mlobeli-Dlakhulu,^27^ the participants revealed that they were often shouted at by health care providers and sent home without help, especially when the counsellors were not around. Their health records were marked with coloured stickers in order to identify their positive HIV status. Aggleton et al.^28^ stated that, from the start of the AIDS epidemic, stigma and discrimination have fuelled the transmission of HIV. These perceptions have greatly increased the negative impact associated with the epidemic, creating major barriers to preventing further infection, alleviating impact and providing treatment, adequate care and support.

Participants expressed negative experiences with regard to the pill count practices. They appreciated the general cleanliness of the environment of the ART clinic. They verbalised their feelings, however, about a disgusting practice made by nurses to count their medications using unwashed hands and then expecting them to take these pills at home. They felt that the infection control principles were not considered because they felt that nurses were contaminating their pills. Participant 2 (female, 32 years, post-secondary education and employed) had this to relate:

‘One day the sister put my pills down on the table, started counting using her hands unwashed, and then tells me, you should start by taking these old ones before the new ones. Some of the pill fell onto her lap and she took them and continued as if there was nothing wrong. I felt it very disgusting that I have to swallow them when I reach home.’

Participant 6 (female, 40 years, post-secondary education and unemployed) showed how he had observed a nurse merely taking a piece of paper and counting his pills without following the correct procedure:

‘I felt bad when my pills were put in a piece of paper and counted using bare hands, why don’t they use plastic/wooden things to count them.’

Pill counts are often used to measure the adherence of the patients to treatment.^29^ According to WHO,^30^ the standard for good dispensing states that using hands is bad practice for both hygienic and product quality reasons. Furthermore, counting should be done using a clean piece of paper and a clean knife or spatula, a clean tablet counting device, the lid of the stock container and or any other clean dust-free surface.

## Discussion

The positive patients’ experiences with the quality of care and patient satisfaction with the clinic are considered to be important elements in quality improvement work at the facility and are also seen as indicators of the quality of health care.^31^ Participants were satisfied with the positive attitudes of the health professionals at the ART clinic. The clean environment, which was said to be safe and at less risk to infection, was also mentioned. Receiving care by competent health care personnel comprised being listened to, treated individually and not as one amongst many. Wagner and Bear^32^ indicated that patients who are satisfied with their nursing care are more likely to follow treatment and, consequently, have better health outcomes. This is important in the context of this study, in order to retain the HIV-positive clients in the programme and to achieve undetectable viral load. Patients’ experiences of quality of care affect also their health-seeking behaviour after discharge, and when revisiting the same ART clinic.^33^ Patient satisfaction is also an important contributor to both a physical and mental health-related quality of life.^34,35^

Participants wanted to feel valued by having their difficulties appreciated and understood by others. Stigma seems to play a role at this workplace, and has become a source of fear of catching the virus. The findings of the study by Pendukeni^36^ indicated that professional nurses may contract the virus if they do not always remember to take precautionary measures. This was supported by De Villiers and Ndou^37^ when indicating that health care workers were reluctant to provide HIV and AIDS care as a result of concerns about occupational infection and who said that they experienced stress. The health care provider must take precautionary measures to protect themselves, but not to stigmatise or discriminate against the patient. The feeling of not being understood in many cases contributed to the participants’ low sense of personal value.

Nurses are confronted with organisation policies that are focused on cost-efficiency, transparency and accountability goals. Hence, patient care requires well-trained nurses who are capable of creating a safe and patient-centred environment. During the clinic visits, health providers are trained to count the number of anti-retroviral pills left in a pill bottle and to counsel the patient about adherence if the patient missed dosages. This process should be done in consideration of all the protocols, standard operating procedures and the principles of infection control, which was lacking in this study.

### Recommendations

The research will help health professionals, clinicians and policy makers understand better the barriers to high quality care. Such knowledge will help in the development of interventions that could help patients and health professionals establish more stable and functional relationships that could result in better quality care. The following activities should be strengthened by ensuring that:

- Nurses practice correct procedures in caring for all patients without discriminating against those who are HIV-positive.
- Provision is made for pill counting devices such as plastic boards and spatulas in all clinical facilities providing ART.
- Hand washbasins with water and soap are provided. Health professionals must ensure that all the staff follow the hand washing procedure correctly. They should also ensure that there are minimal or no interruptions during counselling sessions by using appropriate notices on the doors that bar interruptions.

## Conclusion

Perceptions of HIV-positive patients of care described by health professionals at the ART clinic were explored. Data were collected from 25 participants through in-depth individual interviews. Participants expressed positive experiences with regard to the environment and the attitudes of health professionals and negative experiences with regard to the practices by health care providers. The patients’ perceptions of the quality of care and their satisfaction with this may affect health outcomes. This could lead to positive perceptions, compliance and adherence to ARV treatment. Health care professionals need to adhere to the principles of infection control.
